# Spectrum of thrombotic complications and their outcomes in Chinese children with primary nephrotic syndrome

**DOI:** 10.1186/s13052-020-00942-0

**Published:** 2020-12-09

**Authors:** Yan-Li Lv, Na Guan, Jie Ding, Yong Yao, Hui-Jie Xiao, Xu-Hui Zhong, Fang Wang, Xiao-Yu Liu, Hong-Wen Zhang, Bai-Ge Su, Ke Xu

**Affiliations:** 1 Department of Pediatrics, Peking University First Hospital, Beijing, China; 2 Department of Pediatrics, Changzhi Medical College, Changzhi, Shanxi China

**Keywords:** Nephrotic syndrome, Child, Thrombosis

## Abstract

**Background:**

Thromboembolism is a life-threatening, limb-threatening or organ-threatening complication that occurs in patients with primary nephrotic syndrome (NS). There are few studies on the spectrum, complications and outcomes of thrombosis in children with NS. This study aimed to determine the spectrum of thrombosis and its relationship with the nephrotic state, treatment and outcomes in children and adolescents with primary NS.

**Methods:**

The medical records of subjects aged 1–18 years with NS complicated with thromboembolism treated at our centre within the last 26 years were retrieved. Data on the status of NS, site, symptoms and signs, laboratory investigations, diagnosis, treatment, complications and outcomes of thrombosis were collected and reviewed retrospectively. A severe complication was defined as a condition associated with thrombosis requiring a special diagnostic modality to confirm or a specific treatment such as surgical intervention. The outcome of thrombosis was defined as the status of thrombosis, as determined by imaging methods and the functional status with respect to the anatomic sites of thrombosis at the last follow-up. The permanent dysfunction of an organ or limb related to thrombosis was defined as a sequela.

**Results:**

We observed thrombosis in 1.4% (27/1995) of subjects with NS during the study period. There were 27 subjects with thrombosis, including 21 males and 6 females. Thrombosis was observed in 51.9% (14/27) of the study participants with steroid resistant NS. Most episodes of thrombosis occurred during the active stage of NS; however, 7.4% of thrombosis cases occurred during the remission of proteinuria. Renal vein thrombosis (33.3%) and pulmonary embolism (25.9%) were the most common types of thrombosis. Among the 17 subjects biopsied, minimal change disease and membranous nephropathy were the two most common findings. Six (22.2%) subjects experienced severe complications or sequelae; 1 had persistent intracranial hypertension, 1 had intestinal perforation, 1 had hypoxemia and pulmonary hypertension, 1 had lameness, 1 had epilepsy, and 1 had an askew mouth due to facial paralysis. In 19 (70.4%) subjects, the symptoms resolved completely or improved without severe complications or sequelae.

**Conclusions:**

Thrombosis mostly occurred in males of school age during the active stage of NS. Renal vein thrombosis and pulmonary embolism were the most common types of thrombosis. In most patients with thrombosis, the symptoms improved completely without complications with standard anticoagulation therapy. However, 22.2% had severe complications or sequelae requiring an advanced diagnostic modality and aggressive treatment.

## Background

Primary nephrotic syndrome (NS) is a common renal disease in children. Children with NS present with oedema, heavy proteinuria, hypoalbuminemia and hyperlipidaemia [1]. Thromboembolism is a rare but life-threatening or organ-threatening complication occurring in children with NS [2–8]. Thrombosis occurs mostly in veins and rarely in arteries. Thrombosis can occur in any vessel in patients with NS, but the most common sites are the deep veins of leg (DVL), renal vein (RV) and pulmonary artery [9–14]. The underlying pathophysiology of thrombosis mainly includes the patient’s susceptibility to thrombosis, an NS-related hypercoagulable state and treatment-related risks, such as central venous catheters and diuretics [5, 8]. Imaging methods such as Doppler ultrasonography (DUS), magnetic resonance imaging (MRI) and computerized tomography (CT) are commonly used for the diagnosis of thrombosis. From the onset of thrombosis until the disappearance of thrombosis, thrombosis can delay the treatment of NS, prolong the patient’s hospital stay time, threaten the affected organ and even lead to death. Death due to pulmonary embolism (PE), superior vena cava thrombosis, cerebral arterial thrombosis, and dural sinus thrombosis in children with NS has been reported [2, 15, 16]. Loss of vision, necrosis of the small bowel and coronary syndrome due to thrombosis have been reported [10, 12, 14]. Once thrombosis is diagnosed, therapeutic anticoagulation therapy lasting approximately 3 months followed by prophylactic anticoagulation therapy until NS is in remission is recommended [5, 17]. For life-threatening thrombosis or organ-threatening thrombosis, thrombolysis or thrombectomy is recommended [17]. The spectrum of thrombosis in children with NS differs across centres. Few studies have been conducted on the spectrum, associated complications and outcomes of thrombosis in children with NS [2, 6, 15]. The objective of our study was to determine the spectrum of thrombosis and its relationship with the nephrotic state, treatment and outcomes in children and adolescents with primary NS.

## Methods

### Patients

This is a retrospective study performed in the Department of Paediatrics of Peking University First Hospital of China. The medical records of subjects with NS treated from January 1993 to March 2019 were retrieved from the medical record system and screened according to the discharge diagnosis codes. Subjects aged 1 to 18 years with NS complicated with thrombosis were included. NS was defined by the standard definition (24-h urinary protein  ≥50 mg/kg body weight and serum albumin < 25 g/L) stated in the Chinese Medical Association guidelines. Only subjects with NS who were diagnosed with thrombosis by imaging examinations were included. Subjects with congenital or genetic NS, secondary NS, or IgA nephropathy were excluded. This study was approved by the Ethics Committee of Peking University First Hospital.

### Data collection

**Fig. 1:**
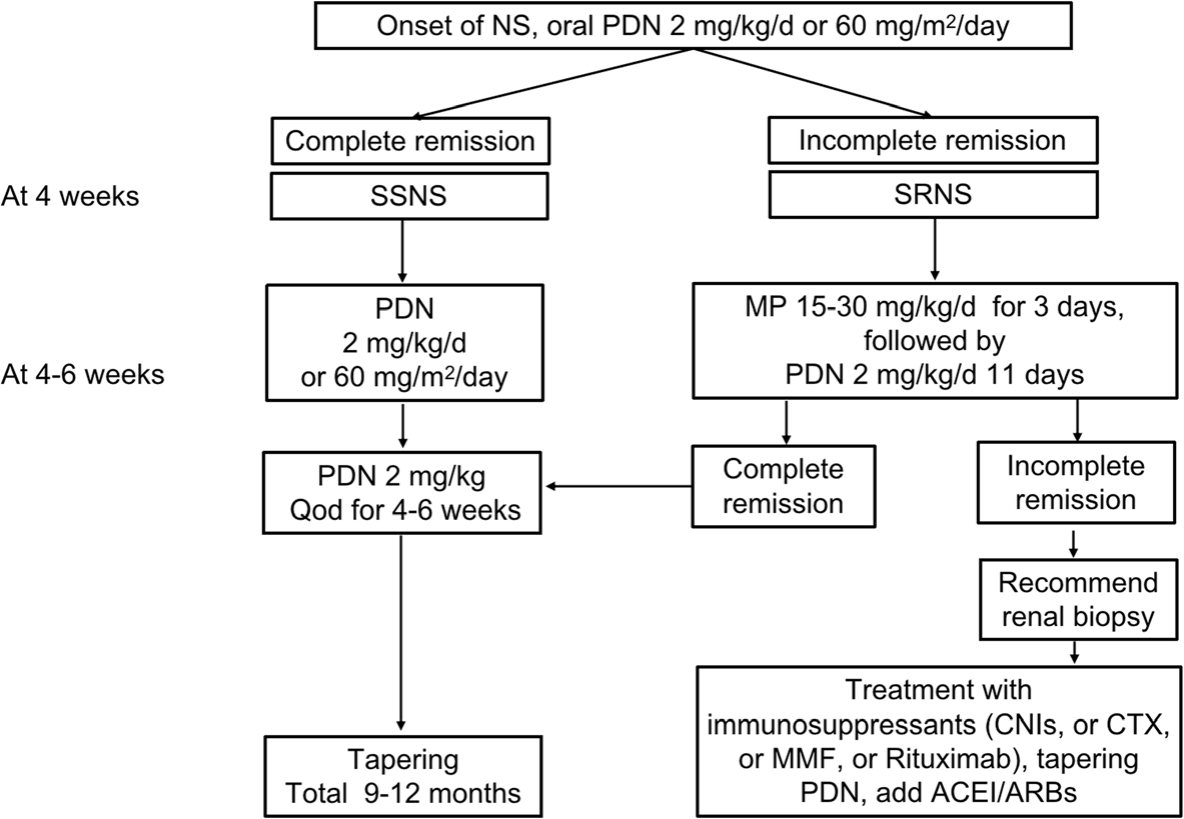
Flow chart of the diagnostic and treatment processes for NS. NS, nephrotic syndrome; PDN, prednisone/prednisolone; SSNS, steroid sensitive nephrotic syndrome; SRNS, steroid resistant nephrotic syndrome, MP, methylprednisolone; CNIs, calcinurin inhibitors; CTX, cyclophosphamide; MMF, mycophenolate mofetil; ACEI/ARBs, angiotensin converting enzyme inhibitor/angiotensin receptor blockers

The data collected included demographic information, the possible risk factors for thrombosis, such as a recent history (within 1 week before the diagnosis of thrombosis) of vomiting or diarrhoea, infection, renal insufficiency, central venous catheterization, a past history of thrombosis and a family history of thrombosis, and the status of NS at the onset of thrombosis, such as first episode or relapse, steroid sensitive NS (SSNS) or steroid resistant NS (SRNS), and immunosuppressive therapy. A flow chart showing the diagnostic and treatment processes for NS is shown in Fig. 1. SSNS was defined as the absence of oedema and proteinuria on 3 consecutive days within 4 weeks of oral prednisone/prednisolone therapy at dosages of 2 mg/kg/d or 60 mg/m^2^/d, as stated in the Chinese Medical Association guidelines. If the patient did not achieve complete remission within 4 weeks of prednisone/prednisolone therapy at dosages of 2 mg/kg/d or 60 mg/m^2^/d, the patient was considered to have SRNS. Complete remission and relapse were defined according to the guidelines [18]. Heavy proteinuria was defined as a urinary protein level ≥ 3+ or 24-h urinary protein level  ≥50 mg/kg, and mild or moderate proteinuria was defined as a urinary protein level of 1+ to 2+ by dipstick or a 24-h urinary protein level of > 150 mg/d and < 50 mg/kg. The information retrieved on thrombosis included the site, symptoms, signs, platelet count, fibrinogen level, plasma D-dimer findings, anti-thrombin-III level, protein C level, protein S level, antiphospholipid antibody level, lupus anticoagulant level, homocysteine level, imaging findings on DUS, CT or MRI and treatment (heparin or low molecular weight heparin with duration, warfarin), thrombosis-associated complications and outcome of thrombosis. The thrombosis-associated complications were defined as death or dysfunction of the corresponding site caused by thrombosis within 3 months from the onset of thrombosis that required a special diagnostic modality to confirm or a specific treatment, such as surgical intervention. The outcome of thrombosis was defined as the status of thrombosis, as determined by imaging methods and the functional status related to the anatomic sites of thrombosis at the time of the last follow-up. The permanent dysfunction of an organ or limb related to thrombosis was defined as a sequela.

### Statistical analysis

SPSS 20.0 software (SPSS, USA) was used for analysis. A descriptive method was used. The data are expressed as the means ± standard derivations or medians (min, max), depending on the normality of the data.

## Results

A total of 2493 subjects with NS were admitted during the study period. Among them, 498 were excluded (176 had IgA nephropathy, 2 had post-streptococcal glomerulonephritis, 149 had secondary NS, and 171 had infantile or genetic NS). Therefore, data from 1995 subjects with primary NS were analysed. Among these subjects, 27 (1.4%), including 21 males and 6 females, had complications of thrombosis. None of them had a past history or family history of thrombosis. The mean age of NS onset was 7.9 ± 4.4 years, and the age of thrombosis onset was 9.5 ± 4.0 years. The duration of NS before the onset of thrombosis was 19.7 ± 33.5 months.

### Status of the children with NS at the time of diagnosis of thrombosis

Among the 27 subjects, 14 (51.9%) had SRNS, and 9 had SSNS (Table 1). At the onset of thrombosis, 2 subjects did not undergo therapy with corticosteroid and immunosuppressive agents, 25 subjects underwent therapy with corticosteroids, and 7 subjects underwent additional therapy with immunosuppressants (4 used calcinurin inhibitors, 2 used mycophenolate mofetil, and 1 used cyclophosphamide). Before the onset of thrombosis, 25 subjects had persistent proteinuria lasting 67 ± 56 days [median 49 (4, 200)]. Twenty-three of 27 (85.2%) subjects had heavy proteinuria, 2 had mild or moderate proteinuria, and 2 had trace or negative urinary protein levels. Two subjects developed thrombosis between 6 and 7 days after the remission of NS. The associated risk factors are shown in Table 1. Renal biopsy was performed before the onset of thrombosis in 10 subjects and after the onset of thrombosis in 7 subjects. The renal pathological changes included minimal change disease (MCD) in 7 (41.2%) subjects, membranous nephropathy (MN) in 5 (29.4%) subjects, focal segmental glomerulosclerosis in 3 subjects and mesangial proliferative glomerulonephritis in 2 subjects. Before the diagnosis of thrombosis, 13 subjects underwent prophylactic anticoagulation therapy.

**Table 1:** Status of children with nephrotic syndrome at the time of diagnosis of thrombosis

Characteristics	N (%)
Steroid response	
Steroid sensitive	9 (33.3%)
Steroid resistant	14 (51.9%)
Unknown^a^	4 (14.8%)
Episode of NS	
Initial	16 (59.3%)
Relapse	11 (40.7%)
NS status	
Active state	25 (92.6%)
Remission	2 (7.4%)
Associated risk factors	
Infection	11 (40.7%)
Vomiting or diarrhoea	13 (48.1%)
Central venous catheter	7 (25.9%)
Renal insufficiency	5 (18.5%)
Hypotension	1 (3.7%)

^a^The steroid response was unknown because the children did not receive corticosteroid therapy for at least 4 weeks

### Clinical spectrum of thrombosis in children with NS

There were 24 subjects with venous thrombosis and 3 with arterial thrombosis. Twenty subjects had thrombosis at a single site, whereas 7 subjects had thrombosis at multiple sites.

Among the sites of venous thrombosis (Table 2), the RV (9/27, 33.3%) was the most common site, followed by the lungs (7/27, 25.9%), DVL (6/27, 22.2%), cerebral venous sinus (CVS) (6/27, 22.2%), inferior vena cava (IVC) (3/27, 11.1%), portal vein (PV) (2/27, 7.4%), superior mesenteric vein (SMV) (2/27, 7.4%) and internal jugular vein (IJV) (1/27, 3.7%). The various manifestations are shown in Table 2 according to the site of thrombosis. The laboratory findings are shown in Table 3.

**Table 2:** Clinical spectrum of thrombosis in children with nephrotic syndrome

Sites of thrombosis	N	Symptomatic (n)	Manifestations
Renal vein	9	5	Abdominal pain, gross haematuria
Pulmonary artery	7	5	Chest pain, shortness of breath, polypnea and haemoptysis
Deep veins of leg			
CVC-related	6	5	Swelling of the leg
Non-CVC related	0		
Cerebral venous sinus	6	6	Headache, vomiting, transient loss of vision, irregular breathing
Inferior vena cava	3	0	None
Portal vein	2	1	Abdominal pain, diarrhoea and vomiting
Superior mesenteric vein	2	2	Refractory ascites, abdominal pain, diarrhoea and vomiting
Internal jugular vein	1	1	Neck pain
Cerebral artery	2	2	Convulsions, headache, facial paralysis, limb dyskinesia
Popliteal artery	1	1	Leg pain, numbness, swelling and lameness

*CVC* central venous catheterization

**Table 3:** Specific diagnostic laboratory investigations in children with thrombosis (*n* = 27)

	Number of patients/number of patients tested	
	n/n	%
Thrombocytosis (platelets > 450*10^9/L)	4/27	14.8
High fibrinogen (Fibrinogen > 4 g/L)	13/27	48.1
High D-dimer (D-dimer > 0.5 mg/L)	21/26	80.8
Low anti-thrombin-III (anti-thrombin-III < 80%)	6/7	85.7
Low Protein C (Protein C < 76%)	1/7	14.3
Low Protein S (Protein S < 69%)	7/7	100
High homocysteine (homocysteine > 16 μmol/L)	3/10	30.0
Anticardiolipin antibodies positive	0/11	0.0
Anti-β 2 glycoprotein positive	1/4	25.0
Lupus anticoagulant positive	3/9	33.3

### Effects of thrombosis/complications

From the onset of thrombosis until the disappearance of thrombosis, no subjects died of thrombosis, and complications were detected in 4 (4/27, 14.8%) subjects. Intestinal necrosis and intestinal perforation occurred in 1 subject with thrombosis in the PV, SMV and splenic vein. Persistent intracranial hypertension occurred in 1 subject with CVS thrombosis. One subject with PE had an arterial blood oxygen saturation level < 95%; he needed oxygen at 1 L/min from a nasal catheter to maintain an oxygen saturation level > 95%. He had pulmonary hypertension [mean pulmonary arterial pressure of 48.7 mmHg]. Twenty days later, his mean pulmonary arterial pressure decreased to 31.6 mmHg. Lameness occurred in 1 subject with popliteal arterial thrombosis.

### Diagnostic imaging findings

When patients had the manifestations shown in Table 2, such as pain or swelling in a specific location or an increased D-dimer level, thrombosis was suspected, and imaging methods such as DUS, MRI and CT were used for the diagnosis of thrombosis. The imaging methods used to diagnose thrombosis and their findings are shown in Table 4, and DUS was the most commonly used method.

**Table 4:** Imaging findings in children with thrombosis

Sites	Total	Imaging diagnosis method				
		DUS	CT	MRV	MRI	Pulmonary ventilation/perfusion imaging
Renal vein	9	5	4			
Pulmonary artery	7		4			3
Deep veins of leg	6	6				
Cerebral venous sinus	6			6		
Inferior vena cava	3	3				
Portal vein	2	2				
Superior mesenteric vein	2		2			
Internal jugular vein	1	1				
Cerebral artery	2				2	
Popliteal artery	1	1				

*CT* computed tomography, *DUS* Doppler ultrasonography, *MRV* magnetic resonance venography, *MRI* magnetic resonance imaging

### Treatment of thrombosis

After the diagnosis of thrombosis, 2 subjects left the hospital voluntarily, and 25 subjects received standard anticoagulation therapy with heparin or low-molecular-weight heparin first, followed by warfarin for a planned duration of 3–6 months, depending on the sites of thrombosis and the NS status of the subject [17]. Heparin was given in a bolus dose of 75–100 U/kg, the maintenance doses were 10–20 U/kg per hour, and the activated partial thromboplastin time was monitored (target 60–85 s). Low-molecular-weight heparin was given in doses of 100 U/kg every 12 h subcutaneously, and anti-Xa level (target 0.5–1.0 U/ml) was monitored. Warfarin was initially given in a dose of 0.1–0.2 mg/kg (< 5 mg), and the dosage was adjusted according to the international normalized ratio (target 2.0–3.0). Within the follow-up period, the subjects received anticoagulation therapy for 4.4 ± 3.9 months. Seven subjects underwent thrombolysis.

In addition, 1 patient with intestinal perforation underwent laparotomy, end-to-end anastomosis, and an abdominal operation and fully recovered. One patient with persistent intracranial hypertension underwent a lumbar cistern-peritoneal shunt operation, and his condition improved. One patient with popliteal arterial thrombosis failed to show improvement after anticoagulation therapy and thrombolysis. She underwent embolectomy successfully.

### Outcomes of thrombosis

The outcomes of thrombosis at different sites are shown in Table 5. In nineteen (70.4%) subjects, the symptoms resolved completely or improved without complications or sequelae. In three subjects who experienced severe complications, including 1 with intestinal perforation, 1 with persistent intracranial hypertension and 1 with pulmonary hypertension, the symptoms resolved completely without sequelae. Three (3/27, 11.1%) subjects developed sequelae; among them, 1 subject with intracranial arterial thrombosis had epilepsy, 1 subject with intracranial arterial thrombosis had an askew mouth (due to facial paralysis), and 1 subject with popliteal arterial thrombosis had lameness. Two subjects were lost to follow-up. Recurrent thrombosis was observed in 1 subject who had CVS thrombosis associated with the relapse of NS.

**Table 5:** Treatment and outcomes of thromboembolism in children with primary nephrotic syndrome

Sites	Number of patients followed-up/total patients	Follow-up duration, median (min, max days)	Serious complication (n)	Outcomes of thrombosis			
				D (n)	I (n)	R (n)	S (n)
RV	8/9	52 (26, 173)	0	7	1	0	0
PA	4/7	100 (76, 128)	1, hypoxemia and pulmonary hypertension	2	2	0	0
DVL	6/6	34 (6, 133)	0	5	1	0	0
CVS	6/6	106 (35, 1095)	1, persistent intracranial hypertension	2	4	1	0
IVC	3/3	102, 50, 76	0	3	0	0	0
PV	1/2	33	0	1	0	0	0
SMV	2/2	33, 54	1, intestinal necrosis	2	0	0	0
IJV	1/1	30	0	0	1	0	0
CA	2/2	25, 85	0	1	1	0	2^a^
PA	1/1	78	1, lameness	1	0	0	Lameness

*RV* renal vein, *PA* pulmonary artery, *DVL* deep veins of leg, *CVS* cerebral venous sinus, *IVC* inferior vena caca, *PV* portal vein, *SMV* superior mesenteric vein, *IJV* internal jugular vein, *CA* cerebral artery, *PA* Popliteal artery, *D* disappearance, *I* improvement, *R* recurrence of thrombosis, *S* sequela

^a^1 epilepsy, 1 askew of mouth due to facial paralysis

## Discussion

This retrospective study described the spectrum, associated severe complications and outcomes of thrombosis in children and adolescents with primary NS treated in our centre within a period of 26 years. The incidence of thrombosis in this study is lower than the reported overall rate of 1.8–6.6% [3–5, 7]. The average onset age (9.5 ± 4.0 years) is similar to those reported by Tavil et al. (7.1 ± 4.9 years) and Suri et al. (7.7 ± 2.7 years) [6, 15]. In contrast to some reports that females had a higher incidence of thrombosis than males in patients with NS, most of the subjects in this study were male [2, 7]. In contrast to Suri’s report that 14.7% of thrombosis events occurred during the first episode of NS, thrombosis in most of the subjects in this study occurred during the first episode of NS [15].

Severe proteinuria has been proposed to be a predictor of thrombosis [7]. Similar to previous reports, in this study, thrombosis occurred in most subjects during the active stage of NS [2, 6]. However, 7.4% of subjects developed thrombosis within 1 week after the remission of NS, including 1 with CVS and 1 with intracranial arterial thrombosis. Suri et al. also reported that 11.4% of children developed thromboembolism during the remission of NS, including 3 with CVS and 1 with intracranial arterial thrombosis [15]. This phenomenon suggests that the risk of thrombosis was not completely relieved immediately after the remission of proteinuria. During NS, the hypercoagulative status is mostly attributed to the urine loss of anticoagulative proteins such as anti-thrombin-III, protein S and protein C and a compensatory increase in procoagulant proteins such as fibrinogen, factor V and factor VIII [5]. We think that time is needed for the recovery of the haemostatic balance after the remission of proteinuria. Studies on the exact duration required for the recovery of the haemostatic balance after proteinuria remission are lacking. Based on the above observation, clinicians should also be aware of the risk of thrombosis in children with NS within 1 week after the remission of proteinuria.

Consistent with Andrew’s report, we observed that the most common types of thrombosis in children and adolescents with NS were RV thrombosis and PE [19]. In contrast to the results of our study, DVL and CVS were the most common sites of thrombosis in reports by Lilova et al. and Suri et al., respectively [2, 15]. Symptoms or signs associated with the anatomic location of thrombosis are useful hints for screening thrombosis. However, some children with RV thrombosis, PE, and DVL and IVC thrombosis did not present any corresponding symptoms. It has been previously reported that asymptomatic PE accounts for 28.1% of cases and RV thrombosis accounts for 25.0% of cases [20]. This finding suggests that careful imaging examinations should be considered, irrespective of the symptoms, for patients at high risk of thrombosis. In addition, subjects with thrombosis in the PV and SMV presented with nonspecific symptoms, including abdominal pain, diarrhoea, vomiting, and refractory ascites. For children with abdominal manifestations, PV and SMV thrombosis should be considered.

The frequency of severe complications and sequelae in our study (22.2%) was slightly higher than that reported by Tavil et al., who showed that 17.6% (3/17) of children with thrombosis, including 1 with cerebral infarct and 2 with PV thrombosis, had sequelae [6]. Lilova et al. reported that sequelae or death occurred in 3 of 9 children with thrombosis [2]. Among them, 1 had postphlebitic syndrome, 1 died of superior vena cava thrombosis and 1 died of cerebral arterial thrombosis. Suri et al. reported 2 deaths due to PE among 34 children with NS [15]. In our study, the anatomic sites of thrombosis related to severe complications were the SMV, CVS, pulmonary artery, intracranial artery and leg artery. SMV thrombosis is a very rare but life-threatening condition. Two subjects < 18 years old were reported to have SMV thrombosis during NS: 1 survived after anticoagulation therapy, and 1 died of bowel necrosis and disseminated intravascular coagulation [21]. In our study, 1 of 2 subjects with SMV thrombosis experienced intestinal necrosis, and he survived after the resection of the necrotic bowel. CVS thrombosis is another severe type of thrombosis [22]. In our study, 1 subject with CVS thrombosis had persistent intracranial hypertension that could not be relieved after standard anticoagulation therapy. The subject had to undergo surgery for the placement of a peritoneal shunt in the lumbar cistern, and this subject’s condition improved. No similar cases have been reported previously [23]. PE has been proposed to be a life-threatening condition [15]. Although no one died of PE confirmed by imaging methods in this study, there were two subjects diagnosed with suspected PE without imaging examinations, according to the medical records: 1 subject experienced sudden death, and the other manifested with dyspnoea and hypoxemia and discontinued therapy. One subject with PE had hypoxemia and pulmonary hypertension.

This study was not designed to identify the risk factors for thrombosis. The possible risk factors proposed by previous reports were observed [3, 4, 6, 7]. The use of central venous catheters is considered the most common risk factor for thrombosis in NS [7, 17]. We found that all subjects with DVL thrombosis had a history of central venous catheterization in the corresponding site. MN is thought to be the most common pathological change in children with NS complicated by thrombosis [4, 7]. In 17 subjects with available pathological data, we found that the main pathological change was MCD (7/17, 41.2%), followed by MN. Considering that the overall incidence of thrombosis was not compared among children and adolescents with different pathological changes, no conclusions could be drawn. Thirteen subjects developed thrombosis despite prophylactic anticoagulation, and no conclusions on the effect of prophylactic anticoagulation could be drawn in this study. In addition, most of the subjects with thrombosis in our study had SRNS (51.9%). This finding is similar to those reported in some previous studies. Lilova et al. reported that the frequency of thrombosis in children with SRNS (3.8%) was higher than that in children with SSNS (1.5%) [2]. Tavil et al. reported that most children with thrombosis had SRNS [6]. Andrew et al. proposed that children with SSNS have a low risk of thrombosis [19]. Recently, the International Paediatric Nephrology Association has suggested thrombophilic screening is performed for SRNS children with risk factors including the use of a central venous catheter, persistent proteinuria of a nephrotic range, and a positive family history of thrombosis [8].

This study has limitations. First, this was a retrospective study, and the outcomes of thrombosis were not regularly assessed. Second, no risk factors for thrombosis were analysed. Third, only subjects with thrombosis confirmed by imaging methods were included in the analysis, which might bias the severe complications findings. In addition, the data on the laboratory investigations of thrombosis risk factors, such as anti-thrombin-III, protein S, protein C, and antiphospholipid antibody levels, are too limited to draw conclusions.

## Conclusions

Thrombosis mostly occurred in male subjects of school age. Although most thrombosis cases occurred during the active stage of NS, thrombosis can occur within 1 week after the remission of proteinuria. The most common sites of thrombosis were the RV and pulmonary artery. After aggressive therapy, 70.4% of the cases of thrombosis resolved completely or improved without severe complications or sequelae. Thrombosis in the CVS, SMV and arteries are life-threatening or organ-threatening conditions that deserve more attention and aggressive therapy.

## Data Availability

All data generated or analysed during this study are included in this published article.

## Abbreviations

NS: Nephrotic syndrome
DVL: Deep veins of leg
RV: Renal vein
CVS: Cerebral venous sinus
IVC: Inferior vena cava
PV: Portal vein
SMV: Superior mesenteric vein
IJV: Internal jugular vein
DUS: Doppler ultrasonography
MRI: Magnetic resonance imaging
CT: Computerized tomography
PE: Pulmonary embolism
SSNS: Steroid sensitive NS
SRNS: Steroid resistant NS
MCD: Minimal change disease
MN: Membranous nephropathy
